# Nanobody-Based Sandwich Immunoassay for Pathogenic *Escherichia coli* F17 Strain Detection

**DOI:** 10.3390/bios13020299

**Published:** 2023-02-20

**Authors:** Asma Dhehibi, Abdelmounaaim Allaoui, Amal Raouafi, Mohammed Terrak, Balkiss Bouhaouala-Zahar, Mohamed Hammadi, Noureddine Raouafi, Imed Salhi

**Affiliations:** 1 Livestock and Wildlife Laboratory (LR16IRA04), Arid Lands Institute (I.R.A), University of Gabès, Médenine 4119, Tunisia; 2 Laboratory of Microbiology, African Genome Centre, Mohammed VI Polytechnic University (UM6P), Lot 660—Hay Moulay Rachid, Ben Guerir 43150, Morocco; 3 Sensors and Biosensors Group, Analytical Chemistry and Electrochemistry Lab (LR99ES15), University of Tunis El Manar, Tunis El Manar 2092, Tunisia; 4 InBioS-Centre for Protein Engineering, University of Liege, B-4000 Liege, Belgium; 5 Laboratory of Venoms and Theranostic Applications (LR20IPT01), Place Pasteur, BP74, Pasteur Institute of Tunis, University of Tunis El Manar, Tunis 1002, Tunisia

**Keywords:** immunoassay, nanobody, *Escherichia coli*, F17A fimbria, fluorescence

## Abstract

Rapid and specific detection of pathogenic bacteria in fecal samples is of critical importance for the diagnosis of neonatal diarrhea in veterinary clinics. Nanobodies are a promising tool for the treatment and diagnosis of infectious diseases due to their unique recognition properties. In this study, we report the design of a nanobody-based magnetofluorescent immunoassay for the sensitive detection of pathogenic *Escherichia coli* F17-positive strains (*E. coli* F17). For this, a camel was immunized with purified F17A protein from F17 fimbriae and a nanobody library was constructed by phage display. Two specific anti-F17A nanobodies (Nbs) were selected to design the bioassay. The first one (Nb1) was conjugated to magnetic beads (MBs) to form a complex capable of efficiently capturing the target bacteria. A second horseradish peroxidase (HRP)-conjugated nanobody (Nb4) was used for detection by oxidizing o-phenylenediamine (OPD) to fluorescent 2,3-diaminophenazine (DAP). Our results show that the immunoassay recognizes *E. coli* F17 with high specificity and sensitivity, with a detection limit of 1.8 CFU/mL in only 90 min. Furthermore, we showed that the immunoassay can be applied to fecal samples without pretreatment and remains stable for at least one month when stored at 4 °C.

## 1. Introduction

*Escherichia coli* was first described in 1885 by the German pediatrician Theodore Escherich as *Bacterium coli*, isolated from a child’s stool sample. Later, in 1919, it was renamed *Escherichia coli* (*E. coli*) [1]. Although part of the commensal microbiota of the mammalian gut, *E. coli* can be associated with many intestinal and extra-intestinal pathologies through the acquisition of virulence factors, mainly adhesins or fimbriae and toxins [2]. For pathogenic strains, adhesion to intestinal epithelial cells is an essential step prior to colonization and invasion [3]. These strains typically produce fimbrial or afimbrial adhesins to bind to host cell receptors. Fimbriae are long filamentous polymeric protein structures located on the surface of bacterial cells. In neonates, pathogenic strains have been implicated in epidemics of diarrhea or septicemia [4].

F17 fimbriae are thin filamentous heteropolymers composed of two major subunits: the major structural subunit F17A, of which about 100 copies are assembled to form the fimbriae structure, and the minor adhesive subunit F17G [5]. Numerous studies have shown that the F17-positive fimbriae of *E. coli* are associated with diarrhea or sepsis in different animal species such as dogs, alpacas, poultry, horses, camels and calves [6,7,8,9]. Fimbrial proteins are promising targets for an immunodiagnostic approach. Indeed, they—the major F17A subunit in particular—are abundant and easily accessible on the cell surface [5].

Conventional *E. coli* detection techniques, such as bacterial culture, enzyme-linked immunosorbent assays (ELISA), and polymerase chain reactions (PCR), are frequently used to diagnose colibacillosis. Although these methods are very specific, they are not well suited for field use. Culture requires one or more days, which may delay the optimal time of treatment and PCR requires experienced technicians and specific equipment, while ELISA has low sensitivity [10]. Therefore, the search for a simple, rapid, and more sensitive detection approach for this pathogenic strain is essential to reduce animal losses. Over the past decades, immunoassays have become a complementary diagnostic platform that allows for high sensitivity testing and improved ability to perform measurements even in complex matrices. As a result, researchers have suggested that this approach can reduce antibiotic use and mortality rates [11]. To develop biosensors for pathogenic bacteria, various signal transmission techniques have been investigated, including electrochemiluminescence, piezoelectric quartz crystal microbalance, and electrochemical approaches [12].

Antibodies and antibody fragments are the most commonly used biorecognition elements in biosensors due to their excellent selectivity and high binding affinity to the target [13]. In 1997, a new approach to antibody engineering was inaugurated with the invention of recombinant camelid single-domain antibodies [14]. Single-domain variable fragments of heavy chain antibodies (VHH) or nanobodies (Nbs) are considered promising therapeutic and diagnostic tools. Thus, with a molecular weight of about 15 kDa, Nbs are readily expressed in microorganisms, stable, versatile, and have high target binding affinities [15]. Their single-chain structure and chemical and thermal stability make Nbs a tool of choice for immunosensor development. VHHs can bind to nanoparticles by conjugation, fusion or coupling [16]. For example, VHHs have been conjugated to gold nanoparticles, fused to polymer nanoparticles, coupled to quantum dots, fused to enzymes, and conjugated to a protein. Additionally, various studies have focused on the development of different immunodetection platforms with Nbs. For example, an Nb-based sandwich immunosensor has been developed to detect prostate cancer biomarkers [17]. El-Moghzey et al. designed a competitive electrochemical immunosensor to detect human exposure to pyrethroid insecticides based on an alkaline phosphatase-conjugated VHH [18]. A VHH-based magnetoassay has been used for the diagnosis of human toxocariasis with a detection limit of 10 pg/mL [19]. Finally, a sandwich-type electrochemical immunosensor has been developed to detect bacterial toxins (*Clostroduim difficile* toxins A and B) by conjugating nanobodies to gold nanoparticles [20].

In this work, we report the selection of novel Nbs directed against the F17A protein of the pathogenic *E. coli* F17 strain and their use in the design of a simple magnetofluorescent sandwich immunoassay. The bioassay was used to directly detect pathogenic *E. coli* F17 in fecal samples from sick and healthy calves once key variables that influenced the analytical response were optimized.

## 2. Materials and Methods

### 2.1. Immunization, Construction of VHH Library and Biopanning

The VHH library was constructed by following previously described protocols [21,22]. Briefly, a healthy camel (*Camelus dromedarius*) was immunized with five injections of the purified F17A protein. Total RNA was isolated from Histopaque 1077 (Sigma, Saint-Louis, MO, USA)-gradient purified peripheral blood mononuclear cells and used to synthesize the cDNA with oligo (dT). A two-step nested PCR amplification was performed to synthesize the VHH genes using the primers CALL001/CALL002 and Fr1For/Fr4Rev (see Table S1 in the Supporting Information). The second PCR product was cloned into the pHEN4 phagemid and used to transform electrocompetent *E. coli* TG1 cells. The transformed cells were cultured and infected with helper phage M13KO7 to construct a VHH-phage display library (see Supporting Information). The F17A-specific phage virions were selected after three consecutive rounds of biopanning on 96-well strip plates (see Supporting Information). Library enrichment of the specific phages was monitored by ELISA with all phages eluted from each panning (polyclonal phage ELISA) and then with 96 randomly selected single phages after the third panning (monoclonal phage ELISA). ELISA-positive phages were sequenced and unique clones were subsequently selected (see Supporting Information).

### 2.2. Subcloning, Nanobody Expression and Purification

Recombinant pHEN4 plasmids purified from positive clones previously selected by monoclonal ELISA were sequenced. Peptide sequences, deduced by bioinformatics prediction, were aligned and clustered according to their CDR3 regions. Candidate VHH genes were subcloned by infusion cloning (Takara, Japan) into the expression vector pET28a (+) with an N-terminal 6His tag using the restriction enzymes NdeI and XhoI. The generated constructs were transformed into Stellar strains (Takara, Kyoto, Japan). After sequencing, four clones were transformed into a Lemo21 (DE3) expression strain. Nb expression was induced by 0.1 M IPTG at 37 °C for 5 h. Bacteria were harvested and suspended in lysis buffer (10 mM Tris pH = 8, 300 mM NaCl, 10 mM imidazole and 8 M urea). Then, the recombinant proteins were purified by fast protein liquid chromatography (FPLC) on an IMAC HiTrap column (Cytiva, Uppsala, Sweden), and a desalting column (Cytiva, Uppsala, Sweden) exchanged the buffer for PBS. The molecular weight and purity of the Nbs were assessed by SDS-PAGE.

### 2.3. VHH Binding by Cell-ELISA

To assess the ability of VHHs to bind to fimbriae on the cell surface, a cell-ELISA (Enzyme Linked Immunosorbent Assay) with live bacteria was performed. Both strains (F17 and BL21DE3) were grown overnight at 37 °C in 10 mL of LB medium (10 g tryptone, 5 g yeast extract (Biokar, Pantin, France) and 5 g NaCl in 1 L of distilled water). Cells were washed with PBS buffer once and then resuspended in 10 mL of this buffer; the OD was measured at 600 nm and the number of cells was deduced (1 OD = 8 × 10^8^ cells/mL). Two microplates were coated overnight at 4 °C with 100 μL per well of each of the purified nanobodies diluted in PBS to a final concentration of 10 μg/mL. Post blocking with 5% skimmed milk in PBS for 2 h at RT, different concentrations (10^4^ to 10^9^ cells diluted in PBS buffer) of the *E. coli* F17 strain [23] and the control strain BL-21(DE3) were added and incubated for 1 h at 37 °C. A rabbit anti-F17A polyclonal antibody [12] diluted to 1/1000 was applied for 1 h at 37 °C. An anti-rabbit-HRP conjugated antibody (Invitrogen, Carlsbad, CA, USA) diluted to 1/5000 in PBS was used for detection. Three washes with PBS buffer were performed after each incubation. Finally, 100 μL of OPD-H2O2 HRP substrate solution (0.4 mg/mL of OPD in 0.05 M phosphate citrate buffer at pH 5 and 30 μL of H_2_O_2_ per 20 mL of substrate solution) was added to each well and the absorption was measured at 450 nm using the MultiSkan sky plate reader (ThermoFisher Scientific, Waltham, MA, USA).

### 2.4. Preparation of Nb1 Immunomagnetic Beads

Nb1-MBs were prepared according to the method described by Campuzano et al. with some modifications [24]. Briefly, 5 μL of MBs-CO2H (0.8 mg/mL) were washed twice with 50 μL MES buffer (50 mM, pH = 6) at 950 rpm for 10 min at 25 °C. The supernatant was removed using a magnet. To activate their carboxyl group, the MBs were suspended in 50 μL of an MES solution containing 25 μL N-(3-Dimethylaminopropyl)-N’-ethyl-Carbodiimide (EDC) (100 mM) and 25 μL N-hydroxysuccinimide (NHS) (100 mM), prepared immediately before use, and incubated for one hour at 25 °C under agitation at 950 rpm. After two washing steps with MES, the activated MBs were suspended in 25 μL PBS and mixed with 25 μL Nb1 (0.36 mg/mL). The mixture was then incubated for one hour under the same conditions. After two washes to remove excess Nb1, non-specific sites were blocked by suspending the MBs-Nb1 in 50 μL of 0.1 M ethanolamine solution for an hour. The MBs were washed twice with Tris (0.1 M) followed by a third wash with PBS.

### 2.5. Conjugation of Nb4 to the Horseradish Peroxidase (HRP)

Nb4 was HRP-labeled for detection using the HRP conjugation kit from Abcam (Cambridge, UK), as directed by the manufacturer. The conjugated product was used directly without purification steps.

### 2.6. The Sandwich Immunoassays

The test samples (purified F17A recombinant protein, bacterial strains (see Table S2 in Supporting Information), or stool samples) were incubated with the capture complex (MBs-Nb1) in MES buffer for one hour at 30 °C and 950 rpm. Subsequently, after two washes with PBS, the resulting complex was incubated with the second detection nanobody (Nb4-HRP) for 30 min at 25 °C and 950 rpm. The detection procedure was performed as follows: the MBs obtained were suspended in 150 μL phosphate-citrate solution (50 mM, pH 5) containing 25 mM OPD and 25 mM H_2_O_2_ and were incubated at RT for 15 min. 50 μL of potassium cyanide (10 mM) was added to the sample to block the peroxidase activity. Alternatively, sodium azide, cystine, ethylenethiourea, hydroxylamine, sodium sulfide, or p–aminobenzoic acid can be used as HRP inhibitors. Finally, fluorescence was measured by exciting the sample at 450 nm and the spectra were collected at the emission wavelength of 550 nm.

### 2.7. Spike and Recovery Assay

Fecal samples were gathered from two healthy camel calves and assayed by PCR for the presence of the *E. coli* F17 strain. The negative samples were then spiked with different concentrations of the *E. coli* F17 strain.

## 3. Results

### 3.1. Library Construction and Selection of F17A-Specific Nanobodies

**Figure 1:**
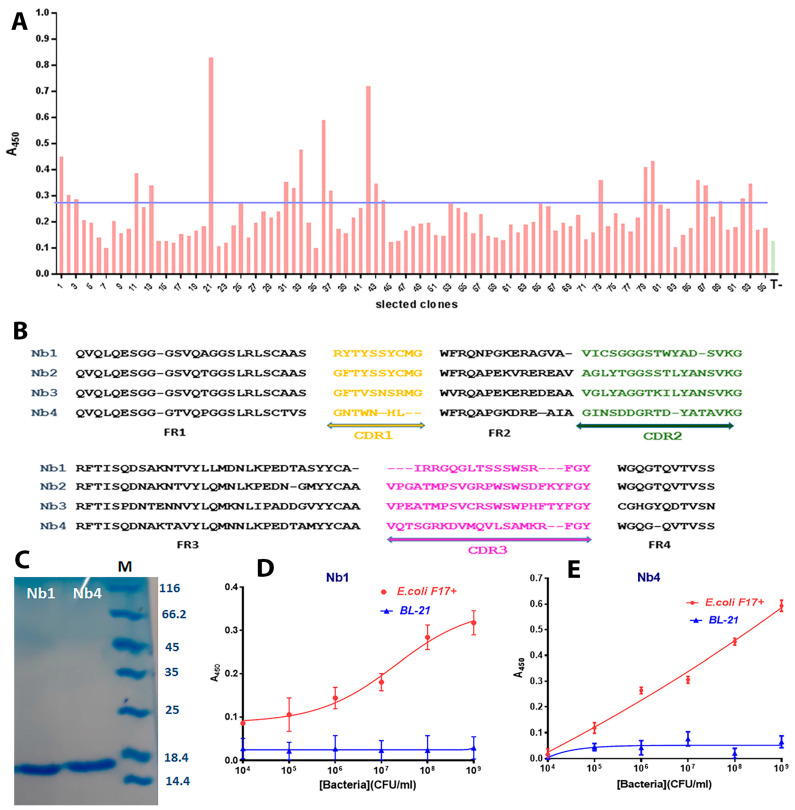
Library screening and nanobody production. (**A**) Monoclonal phage ELISA against the lysate of the F17+ strain. (**B**) Amino acid sequences of the specific anti-F17A nanobodies. CDR designation was performed by a simple alignment with a known nanobody sequence from the Uniprot database. (**C**) SDS-PAGE analysis of purified anti-F17A nanobodies (Nb1 and Nb4) approximately 16 kDa in size. (**D**,**E**) Indirect ELISA test; Nbs against different concentrations (1 × 10^4^ to 1 × 10^9^ CFU/mL) of the *E. coli* F17 and the negative control BL-21 DE3 strains.

Before each antigen injection, blood was collected from the jugular vein and serum was collected post-coagulation by centrifugation. The immune response was monitored by testing the different sera against the F17A protein by ELISA. We showed that the reactivity of the serum increased over the immunization time (Figure S1A). Four days after the last booster, 100 mL of nonclotted blood was collected from which a total of 3 × 10^7^ PBMCs were purified, and total RNA was isolated and converted to cDNA. The first PCR yielded three bands; after gel purification, the 700 bp band (corresponding to the heavy chain antibody genes) was used as a template for the second PCR to amplify the 400 bp VHH genes (Figure S1B). The size of this library in the pHEN4 phagemid was approximately 1 × 10^8^. Twenty-four randomly selected individual colonies were analyzed by colony PCR; over 90% of these clones contained an insert with the appropriate size of a VHH gene (Figure S1C). After 3 rounds of biopanning, the strongest enrichment occurred in the third round, as shown by polyclonal phage ELISA (Figure S1D). The 22 clones selected as positive by the monoclonal phage ELISA against the lysate of the F17-positive bacterial strain were sequenced (Figure 1A).

Analysis of these sequences by bioinformatic tools showed that they belong to four different Nb families sharing the same CDR3 (Cluster of Differentiation) region. The predicted peptide sequences of these Nbs are presented in Figure 1B. The four selected Nbs were subcloned into the expression vector pET28a(+). Nb1 and Nb4 were well expressed and purified by HPLC. After desalting, both recombinant VHHs were highly pure, as deduced from SDS-PAGE (sodium dodecyl sulfate polyacrylamide gel electrophoresis) (Figure 1C). To our knowledge, this is the first report illustrating the production of Nbs against F17A fimbriae. To assess the utility of these Nbs in an immunodiagnostic assay, we performed an indirect ELISA assay to assess their affinity for the target protein in their native functional form on the surface of bacterial cells. For this purpose, we immobilized each Nb by physical adsorption on an ELISA plate and administered varying concentrations of bacterial cells suspended in PBS buffer. The results show that both nanobodies specifically recognize the *E. coli* F17 strain but not the control BL21-DE3 strain. Furthermore, we obtained similar representative sigmoidal curves for each Nb from a minimum bacterial concentration of 1 × 10^4^ CFU/mL and maximum of 1 × 10^9^ CFU/mL (Figure 1D,E).

### 3.2. Design of the Nanobody-Sandwiched Immunoassay

**Scheme 1:**
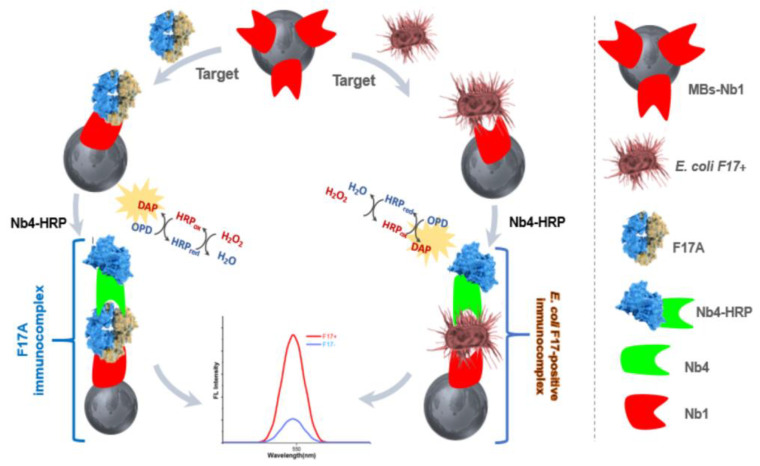
Illustration of the development of the fluorescent bioplatform for the detection of the F17A protein and *E. coli* F17 strain.

To develop an *E. coli* immunoassay, either whole cells or their metabolites are targeted. Since targeting live cells reduces the risk of interference [10], we aimed to develop a sandwich immunoassay capable of detecting the *E. coli* F17 strain in biological samples using our two F17A-specific nanobodies. First, we prepared the MBs-Nb1 capture complex by covalent amide bonding of the terminal amino groups of the nanobody and the carboxylic acid groups of the magnetic beads using EDC/NHS. Then, we applied the target (purified F17A protein or *E. coli* F17 cells). Finally, we added the Nb4-HRP conjugate, and the fluorescence signal was measured in the presence of the enzyme substrate H_2_O_2_ and OPD as the electron donor. To assay the specificity of the immunoassay, fluorescence intensity obtained with and without target was assessed. As shown in Scheme 1, in the presence of F17A or *E. coli* F17, the signal was significantly decreased. These results confirmed the ability of the sandwich immunoassay to specifically detect purified fimbriae and whole bacteria.

### 3.3. Immunoassay Parameter Optimization

**Figure 2:**
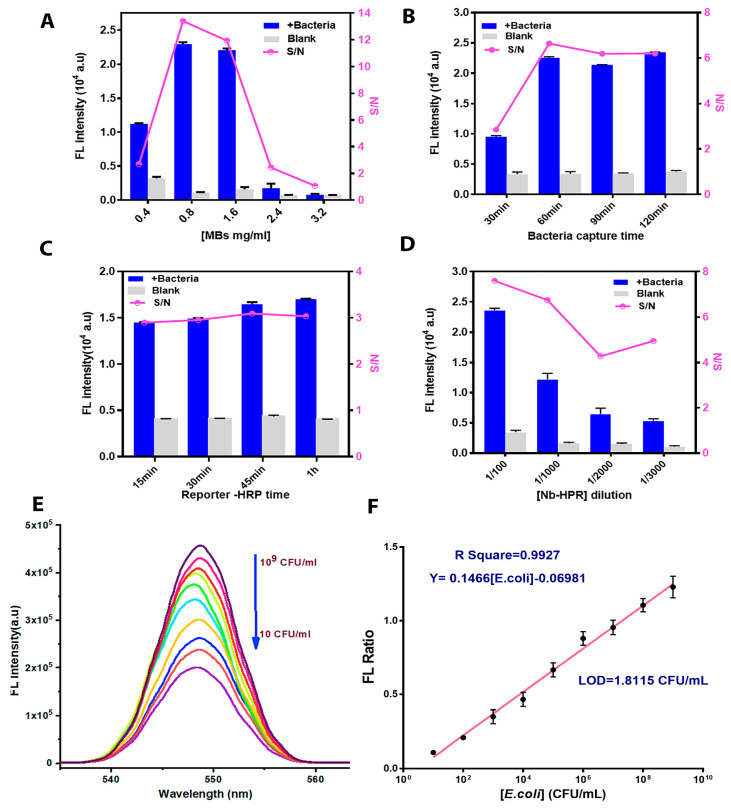
Optimization of the influential parameters of the bioassay. (**A**) Optimization of the MB concentration. (**B**) *E. coli* F17 capture time. (**C**,**D**) Dilution factor of the HRP-conjugated nanobody and its hybridization time. Error bars estimated as three times the standard deviation (*n* = 3). Blue bars (+bacteria): *E. coli* F17 (1 × 10^6^ CFU/mL), grey bars (-bacteria): only PBS solution, S/N: signal-to-noise ratio. (**E**) Fluorescence emission spectra (λ_ex_/λ_em_: 450/550 nm) obtained from various concentrations of F17-positive *E. coli*; (**F**) the corresponding calibration plots. The standard deviations of measurements were calculated from three independent replicates.

To obtain a reliable immunoassay with exploitable analytical performance and sensitive detection of the target bacterium, we optimized various experimental parameters such as MB concentration, bacterial immobilization time, Nb4-HRP concentration and its incubation time. The *E. coli* F17 strain was used as a target in all the optimization tests with a fixed concentration of 1 × 10^6^ CFU/mL. We first examined the effect of the amount of MBs on the validity of the detection by varying the concentration of MBs from 0.4 mg/mL to 3.2 mg/mL. As shown in Figure 2A, the highest S/N ratio was obtained with a concentration of 0.8 mg/mL, and then decreased for higher loadings of magnetic beads. This result can be explained by the decrease in the colloidal stability of the beads and the strict packing of the bio-modified MBs, which decrease the immobilization efficiency of the target [25]. Next, we optimized the capture time of the bacteria by the Nb1-MB complex. As shown in Figure 2B, for one hour and longer, the S/N ratio leveled off. We conclude that 1 h was sufficient to efficiently capture the F17-positive strain. These results could be explained by the presence of a high number of fimbriae, which can facilitate antigen accessibility and adhesion to the exposed Nb1. In addition, the highest fluorescence intensity was obtained at a 1/100 dilution of Nb4-HRP (Figure 2D). Finally, we checked the detection time using Nb4-HRP. The largest S/N ratio was obtained between 15 and 30 min (Figure 2C). For the other parameters, we chose the optimal values reported in literature [24], whereby Nb1 concentration was set at 2.5 μg/mL, conjugation time was set to 1 h, and temperature was set to 25 °C.

### 3.4. Performance Analysis of the Immunoassay

Under optimal conditions, we evaluated the performance of the immunoassay to detect variable concentrations of the F17-positive strain. The emission spectra, shown in Figure 2E, indicate that decreasing the concentration of bacteria from 1 × 10^9^ to 1 × 10^1^ CFU/mL generates a decrease in the intensity of the fluorescence spectra. The obtained calibration curves of the fluorescence ratio (F-F0)/F0 (where, F is the fluorescence intensity in the presence of a target and F0 is the fluorescence intensity in the absence of a target) show a linear relationship as a function of the bacterial concentration with an R-squared equal to 0.9927. We estimated the detection limit (LOD), using the three times “σ/s” criterion (σ, standard deviation of blank; s, slope of calibration curve), with a value of 1.8 CFU/mL (Figure 2F).

The detection limit of this immunoassay is lower than the one provided by ELISA (estimated at 1 × 10^4^ CFU/mL). This suggests that bacterial fimbriae reduce *E. coli* F17 precipitation and binding to the polystyrene surface of the ELISA plate. In our immunoassay, the MBs-Nb1 used can bind to bacterial cells in suspension in all directions. This result suggests that these beads have a higher capture efficiency. To the best of our knowledge, there is no reported immunosensor using nanobodies against the *E. coli* strains, but similar results have been obtained with a sandwich immunoassay based on anti-staphylococcal enterotoxin C nanobodies for the detection of *S. aureus* in milk (LOD = 10 CFU/mL) [26,27]. Furthermore, our current assay showed satisfactory analytical performance compared to other recent conventional immunoplatforms used for the detection of pathogenic *E. coli* (Table 1). Remarkably, the LOD obtained is relatively low compared to those reported in literature. For instance, the developed immunoassay revealed a better LOD than that of an *E. coli* O157:H7 electrochemical sandwich immunoassay (LOD = 1.83 × 10^2^ CFU/mL) based on double-functionalized Au@Pt/SiO2 nanocomposite and immune magnetic nanoparticles [28], or a conductometric *E. coli* sensor (LOD = 1 × 10^4^ CFU/mL) using aptamer-functionalized magnetic beads for bacterial capture [10], or a fiber-optic localized surface plasmon resonance (LSPR)-based sensitive biosensor for detection of *Shigella* bacterial species with a LOD of 1.56 CFU/mL [29]. A pathogenic bacteria sensor based on magnetic nanoparticles [30] and a T4 phage-based biosensor using GOx&HRP-Cu_3_(PO_4_)_2_ for *E. coli* detection in urine [31] had better LODs (1 cell/mL), though in our assay, the bacterial detection time is 90 min instead of the 140 min used in the case of this phage biosensor.

### 3.5. Specificity and Stability of the Sandwich Immunoassay

**Figure 3:**
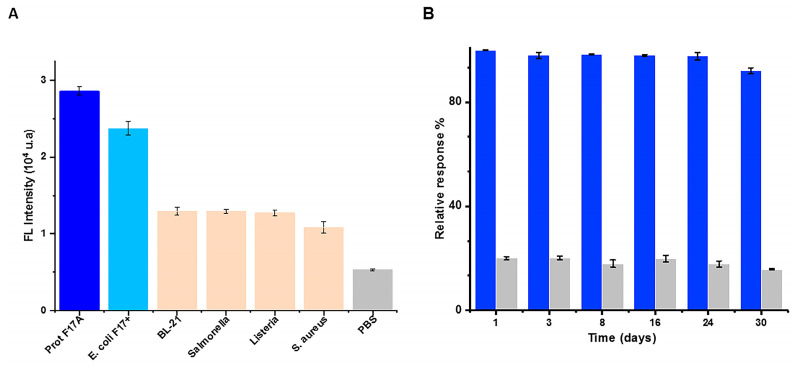
(**A**) Fluorescence intensity for selectivity evaluation obtained with *Salmonella*, *Listeria*, *Staphylococcus*, and *E. coli BL21-DE3*. (**B**) Storage stability of MB-Nb1 based on fluorescence signals measured in the absence and in the presence of the F17-positive *E. coli* strain (*n* = 3).

Antibodies can cross-react with similar proteins and pili shared between several bacterial strains. F17 fimbriae are specific to *E. coli* and a BLAST (Basic Local Alignment Search Tool) analysis on the Uniprot database (uniprot.org (accessed on 15 February 2021) showed only weak identity with the fimbriae of pathogenic bacteria. A cross-reactivity test is essential to evaluate the reliability and the specificity of the diagnostic approach and the specific detection of the pathogenic strain. To assess the specificity of the bioassay, we used four different strains (*E. coli* BL21-DE3, *Salmonella*, *Listeria* and *Staphylococcus*) as non-specific targets with a fixed concentration of 1 × 10^7^ CFU/mL. The results presented in Figure 3A show that fluorescence with *E. coli* F17 was higher compared to the other bacteria, suggesting that the developed immunoassay possesses a higher specificity for the detection of *E. coli* F17. To assess the stability of our assay over time, the capture platform (MB-Nb1) was stored in PBS buffer at 4 °C for 30 days. As shown in Figure 3B, the bioassay retained at least 90% of its initial response, showing great stability. This result can be explained by the excellent stability of Nbs and the strong binding of the Nb to the MBs.

### 3.6. Spike and Recovery Assay

To evaluate the feasibility and applicability of the detection of bacteria in complex biological samples, the developed sensor was tested using two fecal samples that were diluted to 1:100 in PBS and spiked with known concentrations of *E. coli* F17. The specimens were then analyzed as described. As shown in Table 2, the recovery for Sample 1 varied from 89% to 102% and for Sample 2 it varied from 90% to 99%. These results prove the applicability of this nanobody-based immunoassay for specific and rapid detection of *E. coli* F17 in an animal fecal sample.

## 4. Conclusions

In this work, we developed a sensitive magnetofluorescence bioassay for the pathogenic *E. coli* strain expressing the F17 fimbriae based on single-domain antigen binding fragments of camelid heavy chain antibodies (nanobodies). After immunization of a camel with purified recombinant F17A protein, we selected two VHH fragments (Nb1 and Nb4) using phage display. We demonstrated that these Nbs specifically recognize their target on the surface of living bacteria. Both Nbs were further used for the development of the bioassay: Nb1 for capture by conjugation to magnetic beads and Nb4 for detection by conjugation to HRP. Our results show that our biosensor is able to recognize *E. coli* F17 with high specificity and sensitivity, with a LOD of 1.8 CFU/mL in a 90 min assay. We also showed that the biosensor can be used to detect *E. coli* F17 in untreated fecal samples and was still functional after storage at 4 °C for at least one month.

## Figures and Tables

**Table 1:** Performances of reported immunoplatforms for the detection of *E. coli* O157:H7.

Platforms	Antibodies	Linear Range (CFU/mL)	LOD (CFU/mL)	Spiked Real Sample	References
Straptavidin-coated magnetic beads	mAb	10^2^–10^6^	3.9 × 10^2^	Chicken rinse water	[32]
Electropolymerizing PANI on an ITO screen-printed ^a^ electrode	pAb	10^2^–10^6^	10	Tap and River water	[33]
Gold surface	mAb	10^0^–10^5^	10^3^	Milk	[34]
Dendrimer	mAb	3 × 10^0^–3 × 10^5^	3	Meat and Milk	[35]
PPy/AuNPs/MWCNTs/Chitosane hybrid modified pencil graphite electrode (PGE) ^b^	mAb	3 × 10^1^–3 × 10^7^	30	NR	[36]
Poly(amidoamine)dendrimer-encapsulated AuNPs	pAb	10^2^–10^6^	50	Dairy products (Milk, yogurt…)	[30]
Streptavidin-functionalized dynabeads	mAb and pAb	0–500	10^2^	Orange juice	[37]
Gold surface	pAb	10^1^–10^8^	30	Drinking water	[38]
AuNPs/SPCE ^c^	pAb	10^2^–10^9^	37	NR	[12]
Carboxyl Magnetic beads	Nb	10^1^–10^9^	1.8	Fecal sample	This work

^a^: PANI: polyaniline, ITO: indium-doped tin oxide; ^b^: PPy: polypyrrole, AuNPs: gold nanoparticles; NWCNTs: multi-walled carbon nanotubes; PGE: pencil graphite electrode; ^c^: SPCE: screen-printed carbon electrodes; NR: not reported.

**Table 2:** Recovery of *E. coli* in fecal samples.

	*E. coli* Concentration (CFU/mL)		
	Spiked	Detected	Recovery(%)
1	1 × 10^5^ 1 × 10^6^ 1 × 10^7^ 1 × 10^8^	(1.02 ± 0.01) × 10^5^ (0.89 ± 0.001) × 10^6^ (9.19 ± 2.0) × 10^6^ (0.99 ± 0.0008) × 10^8^	102 89 91.9 99
2	1 × 10^5^ 1 × 10^6^ 1 × 10^7^ 1 × 10^8^	(0.99 ± 0.04) × 10^5^ (0.09 ± 1.6) × 10^7^ (0.944 ± 8.8) × 10^7^ (9.4 ± 0.0001) × 10^7^	99 90 94.4 94

## Data Availability

The data presented in this study are available on request from the corresponding author.

## Supplementary Materials

The following supporting information can be downloaded at: https://www.mdpi.com/article/10.3390/bios13020299/s1. Figure S1. (A) ELISA Test presenting the Immune response of camel immunized with F17A purified protein at different times: an increasing immune response was observed after each boost. (B) RNA, PCR 1, PCR 2 and vector profile in 1% agarose gel. (C) Quality control of the library, clones were randomly picked to detect the percentage of clones with a phagemid containing an insert of a proper size for a VHH (PC: positive control, M: 100pb DNA Ladder). (D) Polyclonal phage ELISA: The enrichement for phage particles in wells coated with F17A protein versus wells coated with PBS was detected after each round of paning (Library (Before panning), P1, P2 and P3). All experiments were performed in triplicate; Table S1. sequences of different used primers; Table S2. Molecular biology tools.
