# Supplementary material for: Nanobody-Based Sandwich Immunoassay for Pathogenic Escherichia coli F17 Strain Detection
Source: Biosensors (Basel). 2023 Feb 20;13(2):299. doi: 10.3390/bios13020299 (PMC9953962; doi:10.3390/bios13020299)
Supplement: Supplementary file 1 [file biosensors-13-00299-s001.zip › biosensors-2146124-supplementary.pdf]

*Supplementary Material*

# Nanobody-Based Sandwich Immunoassay for Pathogenic *Escherichia coli* F17 Strain Detection

**Asma Dhehibi [1], Abdelmounaaim Allaoui [2], Amal Raouafi [3], Mohammed Terrak [4], Balkiss Bouhaouala-Zahar [5], Mohamed Hammadi [1], Noureddine Raouafi [3,*] and Imed Salhi [1,*]**

## 1. Apparatuses

Multiskan Sky ELISA plate reader (ThermoFischer Scientific, Waltham, MA, USA) was used to perform all ELISA assays. Fluorescence assay was recorded using a Qubit 4 fluorometer (ThermoFischer Scientific, Waltham, MA, USA). Fluorescence spectra were collected on a PC-controlled SHIMADZU RF-6000 spectrofluorophotometre using a 10 mm cuvette. A Thermo-shaker incubator (ThermoFischer Scientific, Waltham, MA, USA) was used for different incubations of the MB.

## 2. Reagents

Carboxyl modified magnetic beads (MBs) in an aqueous suspension with a diameter of 0.48 µm and a concentration of 10 mg/ml were purchased from New England Biolabs (Ipswich, MA, USA). A horseradish peroxidase (HRP) conjugation kit (ab 102890) was obtained from Abcam (Cambridge, UK). N-(3-Dimethylaminopropyl)-N′-ethyl-Carbodiimide (EDC), N-hydroxysuccinimide (NHS), Sodium citrate (99%) and $H_2O_2$ (30%) were purchased from Sigma-Aldrich (Saint-Louis, MO, USA). Potassium cyanide (KCN, 96%) and o-phenylenediamine dihydrochloride (O-PDA, 98%) were obtained from ACROS Chemicals (Belgium).

**Table S1.** sequences of different used primers.

| Code | Primer sequences |
|---|---|
| F17AFor (NcoI) | 5′CCATGGGCTATGACGTACAAT3′ |
| F17ARev (NotI) | 5′CGAATAGTCATTCGCCGGCG3′ |
| CALL001 | 5′-GTCCTGGCTGCTCTTCTACAAGG-3′ |
| CALL002 | 5′ GGTACGTGCTGTTGAACTGTTCC 3′ |
| FR1for (PstI) | 5′ GATGTGCAG CTGCAGGAGTCTGGGGGAGG 3′ |
| FR4rev (NotI) | 5′ GGACTAGTGCGGCCGCTGGAGACGGTGACCTG GGT 3′ |
| Nb F17A Fr | $^{5'}$GTGCCGCGCGGCAGCCATATGCAGGTGCAGCTGCAGGAG$^{3}$ |
| Nb F17A Rv | $^{5'}$GTGGTGGTGGTGGTGCTCGAGTTAGGAGACGGTGACCTGGGT$^{3'}$ |

**Table S2.** Molecular biology tools.

| Bacteria | Antibiotics | Restriction enzymes | Plasmids |
|---|---|---|---|
| *E. coli F17-positive* | Ampicillin | pstI | pHAT |
| *S. aureus* | Kanamycin | NotI | pHEN4 |
| *Listeria* | | NdeI | Pet28a(+) |
| *Salmonella* | | XhoI | |
| *E. coli BL-21(DE3)* | | | |
| *E. coli JM-109* | | | |
| *E. coli Mach 1* | | | |
| *E. coli Stellar strain* | | | |
| *E. coli Lemo(DE3)* | | | |

## 3. Expresion and Purification of F17A Protein

The cloning, expression and purification of the F17A protein was previously described [1]. Briefly, the bacteria, bearing the recombinant plasmid, were grown for 4 hours at 37°C at 250 rpm in 500 ml of LB medium and the expression of the F17A protein was induced by 1mM IPTG (isopropyl–D-thiogalactopyranoside; Thermo Scientific) for 4 hours. Lysis buffer (8M urea, 500 mM sodium chloride, 10 mM Tris, pH 8.0) was used to harvest and resuspend the cells before being centrifuged for 30 min at 15,000g. The recombinant protein was purified by FPLC on a Hitrap IMAC column (Cytiva, Uppsala, Sweden) with an equilibration buffer (phosphate buffer 20 mM pH 7.4, 500 mM NaCl, 6 M urea and 20 mM imidazole) and an elution buffer (phosphate buffer 20 mM pH 7.4, 500 mM NaCl, 6 M urea and 500 mM imidazole) then renatured by passing through a Hitrap Desalting column (Cytiva) against PBS buffer at pH 7.4. The protein purity was assessed by SDS–PAGE.

## 4. VHH Library Construction and Screening

### *4.1. Camel Immunization*

A dromadary camel was immunized 5 times with 500 μg of purified F17A protein mixed with complete Freund's adjuvant (Sigma) for the first immunization and then with the incomplete adjuvant for the next 4 boosts. Blood samples were collected before each boost to monitor the immune response. Four days after the last boost, a blood sample of 150 mL was collected from the jugular vein of the animal. The lymphocytes were purified on Histopaque 1077 (Sigma) gradient(1/3 Histopaque + 2/3 blood) then centrifuged for 30 min at 400 g without brake at RT.

### *4.2. VHH-phage display library construction*

The VHH library was constructed following the described protocol [2–4]. Briefly, total RNA was isolated by the Trizol (Invitrogen, Carlsbad, CA, USA) method and it was used in cDNA synthesis by reverse transcription with oligo(dT) primers. The obtained cDNA was used in two-step nested PCR reactions, the first targeting the part encoding the variable regions of VHH and VH by a pair of primers CALL001 and CALL002. The product PCR was analyzed by agarose gel electrophoresis and the 700 bp band, corresponding to VHH, was gel purified. The VHH specific genes were amplified by the primers Fr1For and Fr4Rev (containing PstI and NotI sites). After cutting with restriction enzymes, the PCR fragments were cloned into the phagemid vector pHEN4. Thereafter, the ligation product was transformed into electrocompetent *E. coli* TG1 cells and incubate on agar 2YT (with 2% glucose and 100 mg/mL ampicillin) overnight at 37 °C. The next day, the library size was calculated and the colonies were scarped from the plates and stored at −80 °C in 2YT medium supplemented with 50% glycerol. Twenty-four colonies were randomly analyzed by PCR to determine the percentage of colonies with the correct insert size.

### 4.3. *VHH phage display library Screening*

Five hundred microlitres of TG1 bacteria from the library were cultured in 100 mL of 2YT medium containing ampicillin (100 µg/mL) and incubated with shaking at 37 °C for two hours. After that, the bacteria were infected with M13KO7 helper phage and incubated without shaking for 35 minutes at 37 °C. Then, after centrifugation, the pellet was suspended in 300 mL of 2YT medium (100 µg/mL Ampicillin and 70 µg/mL Kanamycin) and incubated overnight at 37 °C with shaking. The phage was precipitated with polyethylene glycol 6000/NaCl (20 %/2.5 M).

The F17A specific phage virions were selected after three consecutive rounds of panning on 96-well strip plates. Purified protein F17A (100 µg) in coating buffer (0.1 M $NaHCO_3$; pH = 8) was used to coat plates at 4 °C overnight. After each round, the phages were eluted with 0.1 M triethylamine and neutralized with 1 M Tris-HCl (pH = 7.4). These output phages were used for the infection of exponentially growing *E. coli* TG1 cells. Then, a part of this culture was used to make a serial dilution and plated on 2YT agar medium (ampicillin/glucose) and the other part for the phages production. After 3 rounds of panning, the enrichment was evaluated with polyclonal phage ELISA.

After the second and third rounds, randomly picked clones were analyzed by monoclonal phage ELISA. Briefly, 190 individual colonies were grown in polystyrene 24-well flat bottom tissue culture plate and incubated with shaking at 37 °C for 3 hours. Subsequently, cells were infected with helper phage. After 30 min without shaking, plates were centrifuged at 3200 g, 10 min at 4 °C. The supernatant was removed and in each well 1.5 mL 2TY medium (Ampicillin/Kanamycin) was added. The microplates were then incubated overnight at 37 °C with shaking. The next day, the phage particles were transformed in two ELISA microplates, previously coated with F17A protein and incubated for 2h at 37 °C. After the application of the anti-M13 phage antibody (1/1000) and the detection by HRP conjugated anti-rabbit antibody (1/5000), the microplates were read at 450 nm. The clones selected as positive were then applied to a plate coated with the lysate of the *E. coli* F17-positive strain, following the same protocol.

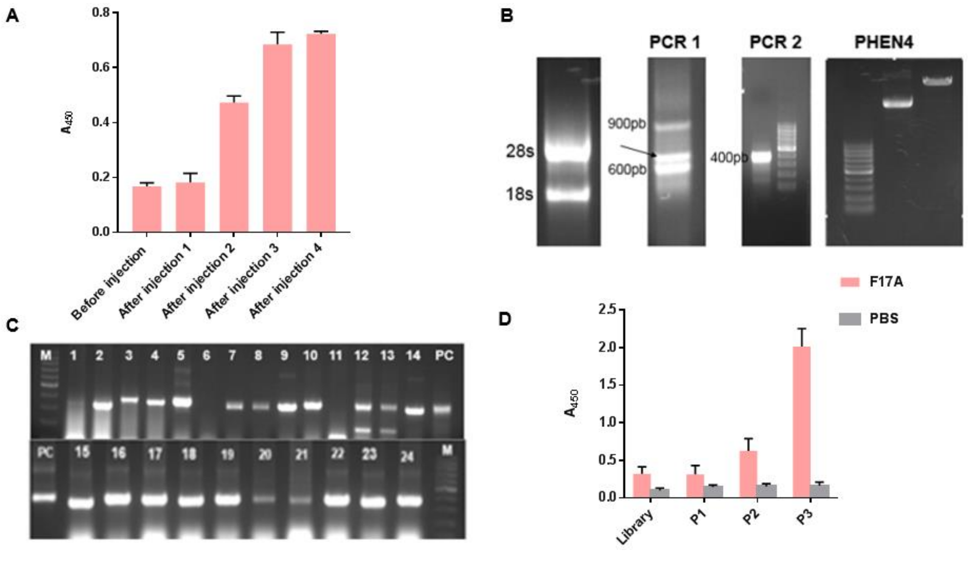


**Figure S1. (A)** ELISA Test presenting the Immune response of camel immunized with F17A purified protein at different times: an increasing immune response was observed after each boost. **(B)** RNA, PCR 1, PCR 2 and vector profile in 1% agarose gel. **(C)** Quality control of the library, clones were randomly picked to detect the percentage of clones with a phagemid containing an insert of a proper size for a VHH (PC: positive control, M: 100pb DNA Ladder). **(D)** Polyclonal phage ELISA**:** The enrichement for phage particles in wells coated with F17A protein versus wells coated with PBS was detected after each round of paning (Library (Before panning), P1, P2 and P3). All experiments were performed in triplicate.

**References**


[1] I. Salhi, A. Rabti, A. Dhehibi, and N. Raouafi, 'Sandwich-Based Immunosensor for Dual-Mode Detection of Pathogenic F17–Positive Escherichia coli Strains', *Int. J. Mol. Sci.*, vol. 23, no. 11, p. 6028, May 2022, doi: 10.3390/ijms23116028.

[2] E. Pardon *et al.*, 'A general protocol for the generation of Nanobodies for structural biology', *Nat. Protoc.*, vol. 9, no. 3, pp. 674–693, Mar. 2014, doi: 10.1038/nprot.2014.039.

[3] T. N. Baral, R. MacKenzie, and M. Arbabi Ghahroudi, 'Single-Domain Antibodies and Their Utility', *Curr. Protoc. Immunol.*, vol. 103, no. 1, Nov. 2013, doi: 10.1002/0471142735.im0217s103.

[4] M. Arbabi Ghahroudi, A. Desmyter, L. Wyns, R. Hamers, and S. Muyldermans, 'Selection and identification of single domain antibody fragments from camel heavy-chain antibodies', *FEBS Lett.*, vol. 414, no. 3, pp. 521–526, Sep. 1997.
